# Semi-surgical percutaneous dilatational tracheostomy vs. conventional percutaneous dilatational tracheostomy: A prospective randomized trial

**DOI:** 10.22088/cjim.12.3.249

**Published:** 2021-04

**Authors:** Novin Nikbakhsh, Fatemeh Amri, Mahmood Monadi, Parviz Amri, Ali Bijani

**Affiliations:** 1 Department of Surgery, Babol University of Medical Sciences, Babol, Iran; 2 Student Research Committee, Babol University of Medical Sciences, Babol, Iran; 3 Department of Internal Medicine, Babol University of Medical Sciences, Babol, Iran; 4 Clinical Research Development Unit of Ayatollah Rouhani Hospital, Babol University of Medical Sciences, Babol, Iran; 5 Mobility Impairment Research Center, Babol University of Medical Sciences, Babol, Iran

**Keywords:** Intensive care unit, Percutaneous dilatational tracheostomy, Complications

## Abstract

**Background:**

Percutaneous dilatational tracheostomy (PDT) is a common surgical procedure in the ICU. The present study was conducted to compare semi-surgical percutaneous dilatational tracheostomy (SSPDT) with conventional percutaneous dilatational tracheostomy (CPDT).

**Methods:**

The present randomized clinical trial was conducted on 160 patients hospitalized in the medical intensive care units (ICUs) with an indication for tracheostomy and were systematically divided into two equal groups of 80. In the CPDT group, after a small incision, a 16-gauge needle was blindly inserted into the trachea and the guidewire was placed inside the lumen. A stoma was created by passing a single dilator over the guidewire. In the SSPDT group, a transverse incision (2 cm) was made 1 cm below the cricoid, and the tracheal ring was then fully reached by releasing the subcutaneous tissues using the index figure, and PDT was then performed. The two groups were compared in terms of their tracheostomy complications (including bleeding, pneumothorax, stoma infection and accidental decannulation) and duration of the procedure.

**Results:**

The two groups were homogeneous in terms of age, gender, mean APACHE score (P>0.05). There were no significant differences between the two groups in terms of the mean time from tracheal intubation to tracheostomy (P=0.869). The duration of the procedure was 5.16±1.72 minutes in the SSPDT group and 6.42±1.71 in the CPDT group (P<0.001). The complication rate was 7(8.75%) in the SSPDT group and 16(20%) in the CPDT group (P=0.043).

**Conclusion:**

SSPDT is safer and has fewer complications than CPDT in ICU patients.

Tracheostomy is a common surgical technique in seriously-ill patients. The indications for tracheotomy include prolonged mechanical ventilation, the need for frequent pulmonary suctioning and reduced consciousness (1-4). Tracheostomy is performed in two ways: Open tracheostomy and percutaneous dilatational tracheostomy (PDT), the latter of which was introduced in 1985 by Ciaglia et al.to make use of bronchoscopy (5). In 2000, Byhahn et al. introduced a modified version of the Ciaglia method called Ciaglia Blue Rhino (CBR). CBR is currently the most common technique used for tracheostomy. This technique uses the Ciaglia single-dilator method and therefore causes less damage to the posterior wall, less bleeding during the operation and less oxygen desaturation. 

PDT is an easy to learn technique and those who have been systematically trained for it can perform it effectively (6-8). The advantages of percutaneous dilatational tracheostomy include smaller cutaneous incisions, simple technique, less tissue damage, less infection and bleeding, fewer transfer complications and reduced costs (4). 

The relative contraindications of PDT include a PEEP higher than 8 cm/H2o and FIo2>50%, history of surgical tracheostomy, unstable cervical spine, uncorrected coagulopathy, cervical mass or previous cervical surgery, increased intracranial pressure and history of mediastina radiation (9). Many studies have confirmed the safety of PDT in short-necked, obese and high-risk patients (10-12). Despite the greater acceptability of conventional PDT compared to surgical tracheostomy, this technique also involves limitations and risks. 

Previous studies have shown that PDT has a prolonged learning curve and may cause more complications if performed by less-experienced healthcare providers (13, 14). PDT complications include tube insertion into the paratracheal space, pneumomediastinum, pneumothorax, subcutaneous emphysema, esophageal rupture, loss of airways, puncture of the tracheal tube cuff, bleeding, stoma site infection, tracheal stenosis, hypoxia, hypotension, accidental insertion of the guide wire into the tracheal tube murphy eye and air embolism (9, 14-17).

In conventional PDT, complications are more likely in cases of difficult anatomy, such as in obese and short-necked patients with tracheal deviation from the midline whose landmarks are not clearly defined. Many other studies have confirmed the safety of PDT without bronchoscopy (14, 18, 19). Various studies have shown the safety of modified tracks under different names, such as modified PDT, hybrid tracheostomy and safe trach with conventional PDT(20- 22). Performing PDT with bronchoscopy has limitations such as interference with lung ventilation (hypoxia and hypercarbia), a prolonged procedure and an increased need for bronchoscopy equipment and an expert operator (23). Ultrasound is also not available in all hospitals and ICUs. Modifying standard PDT in various ways has extended the applicability of PDT without bronchoscopy; however, the complete safety of these changes in standard PDT has not yet been fully demonstrated. 

Based on new studies as well as on the basis of a preliminary study and doing several PDT like surgery (wider incision and exploring soft tissue under the skin), seeing the vessels and preventing severe bleeding and seeing the ring of the trachea, make the SSPDT steps easier to do and fewer complications of vascular and tissue damage.

Given the extensive use of PDT in Iran and in view of the results of earlier studies on the subject, the present study was conducted to compare semi-surgical percutaneous dilatational tracheostomy (SSDT) and conventional percutaneous dilatational tracheostomy (CPDT) in ICUs. 

## Methods

This randomized clinical trial was conducted on ICU patients at Ayatollah Rouhani Hospital of Babol who needed tracheostomy (endotracheal intubation for more than 14 days) from April 2013 to April 2016. The exclusion criteria consisted of emergency tracheostomy, anterior cervical infection, thyromegaly, coagulative disorders, age less than 18 and requiring PEEP>8 and Fio2>50%. This study was performed after the approval of the Research Ethics Committee of Babol University of Medical Sciences with the code MUBABOL.REC.1394.266 and obtaining informed consent from the patient guardians and registering at the Iranian Registry of Clinical Trials with the number IRCT201602297752N8.

 Prior to the start of the study, intensivists were trained by a thoracic surgeon (for 3 months) for wider incision and exploration of the soft tissue. All procedures were performed by one person (intensivist). Thoracic surgeon is an expert in tracheostomy (12 years of experience), and had a supportive role in this study. He has not made any procedure though he was available at the hospital during the procedure. The procedures were performed in the ICU by an intensivist and a trained nurse. Anti-coagulative medications were discontinued 12 to 24 hours before the procedure. The brand name of PDT kit was TRACOE.

Based on the predicted likelihood of surgery complications (10% in the SSPDT group and 30% in the CPDT group), a 20% difference and 80% test power, sample size was estimated as 70 per group; however, it was raised to 80 for a greater assurance. After selecting the patients for tracheostomy, they were divided into one of two groups: CPDT or SSPDT by the method of 40 random blocks with 4 block size (AABB).

Tracheostomy was performed without bronchoscopy; in some cases, however, bronchoscopy was performed to confirm the tracheostomy tube site. Given the possibility of decannulation during the procedure, re-intubation equipment was kept at hand. All the patients underwent mechanical ventilation (ACMV) and were administered oxygen 100% during the procedure and were monitored for their BP (blood pressure), ECG and oxygen saturation (pulse oximeter). Midazolam (0.05mg/kg) and fentanyl (2µg/kg) were administered to the patients. During the tracheostomy, 0.5mg/kg of propofol was administered if required.

The intensivist stood on the right side of the patient and the nurse on the left. The patient’s neck was in the hyperextended position. Five minutes before making the incision, after prepping and draping the anterior cervical area, 3 ml of 2% lidocaine was subcutaneously injected to the skin incision site. 

The tracheal tube cuff deflated 2 ml of air and was pulled back to number 17 in women and number19 in men. In the CPDT group, first, an incision less than 1cm was made approximately in the midline between the cricoid and the sternal notch, and the14-gauge needle was blindly inserted into the trachea and its location was confirmed with air bubble aspiration in the syringe. The guidewire was then placed inside the tracheal lumen, and finally, a stoma was created on the guidewire using the TRACOE single dilator.

In the SSPDT group, all steps were performed similar to CPDT. Except at first, a wider transverse incision (1.5-2 cm) was made in the upper part of the neck, one cm below the cricoid cartilage for easy visualization and easier tracheal access from the skin surface, and the tissues around the trachea were released until the trachea was touched (similar to the surgical tracheostomy). Then a 14-gauge catheter was inserted into the trachea and the remaining stages of the procedure were performed similar to the steps performed in the CPDT group.

A chest x-ray was taken from all the patients to assess their PDT complications and correct the placement of their tracheostomy tube. The two groups were compared in terms of the duration of mechanical ventilation and ICU stay, tracheostomy complications (including bleeding, pneumothorax, stoma infection and accidental decannulation), mortality in the first 24 hours and duration of the procedure (from the time of making the cutaneous incision to the insertion of the tracheostomy tube). Data were analyzed in SPSS using the chi-square test, the paired t-test, the independent t-test, the repeated measures ANOVA and Fisher's exact test, and a P>0.05 was set as the level of statistical significance.

## Results

**Figure 1:**
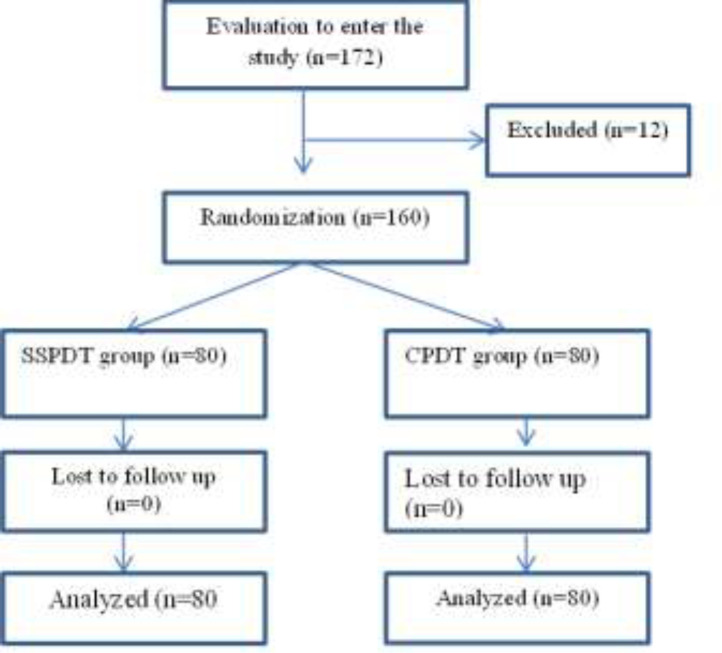
Flow diagram of study protocol

This study was conducted from April 2013 to June 2016 on 172 patients undergoing tracheostomy. Six patients were excluded due to having a BMI>35, one was excluded for a high PEEP, three were excluded for having a short neck and two for thyromegaly. A total of 160 patients were randomly divided into two equal groups of 80 (figure 1).

According to Table 1, cerebrovascular accidents (CVAs) were the most common cause of tracheostomy in the two groups and there were no significant differences between the two groups in terms of diagnosis upon admission (p>0.05).

**Table 1:** The patients' diagnosis upon admission

**P-Value**	**CPDT(n=80) ** **N (%)**	**SSP** **DT(n=80) ** **N (%)**	**Diagnosis**
0.869	8(10) 7(8.8) 48(60) 2(2.5) 2(2.5) 4(5) 6(7.5) 3(3.8)	12(15) 5(6.3) 49(61.3) 2(2.5) 4(5) 3(3.8) 4(5) 1(1.3)	ARF Heart disease CVA Postoperative COPD Sepsis ALS Other

SSPDT: Semi-surgical Percutaneous Dilatational Tracheostomy, CPDT: Conventional Percutaneous Dilatational Tracheostomy, ARF: Acute Respiratory Failure; CVA: Cerebrovascular Accident; ALS: Amyotrophic Lateral Sclerosis; COPD: Chronic Obstructive Pulmonary Disease

According to table 2, no differences were observed between the two groups in terms of age, gender, mean APACHE score, mortality, duration of mechanical ventilation and ICU stay (p>0.05). No significant differences were observed between the groups in the meantime from tracheal intubation to tracheostomy (P=0.869). The most important finding is the duration of the procedure, which is shorter in MPDT group than CPDT group (p<0.001).

**Table 2:** The patient details

**P-Value**	**CPDT n=80**	**SSPDT ** **n=80**	**Variable**
0.638	67.59±16.41	68.74±14.36	Age(year, mean [SD])
0.874	43(53.8)	42(52.5)	Male(male, number [%])
0.475	25.43±5.25	24.81±5.58	APACHE II(mean [SD])
0.992	54.08±16.06	54.05±15.25	Predicted Mortality[SD]
0.316	30(37.5)	24(30)	Actual Mortality[%]
0.869	17.99±8.15	17.75±9.33	Duration of intubation (day, mean [SD])
0.208	26.80±15.23	23.79±11.81	Duration of mechanical ventilation(day, mean [SD])
0.234	32.72±15.90	29.76±12.39	ICU stay(day, mean [SD])
0.108	42(52.5)	52(65)	Success rate(number [%])
<0.001	6.42±1.71	5.16±1.72	Duration of procedure (minutes, mean [SD])

SSPDT: Semi-surgical Percutaneous Dilatational Tracheostomy, CPDT: Conventional Percutaneous Dilatational Tracheostomy, SD: Standard Deviation

According to table 3, the two groups did not differ in terms of stoma infection, hypotension, hypoxemia, bleeding and loss of airways (P>0.05).The complication rate was 7(8.75%) in the SSPDT group and 16(20%) in the CPDT group (P=0.043). In both groups, the complications were mild to moderate and there was no serious complication that caused mortality.

**Table 3:** The complications of conventional SSPDT vs. CPDT

P-Value	CPDT (n=80) N (%)	SSPDT (n=80) N (%)	Variable
1	2(2.5)	2(2.5)	Cuff leak
0.032	7(8.8)	1(1.3)	Multiple attempts at insertion
1	2(2.5)	2(2.5)	Hypotension
0.620	3(3.8)	1(1.3)	Hypoxemia
0.245	3(3.8)	0	Intraoperative bleeding
1	1(1.3)	1(1.3)	Postoperative bleeding
0.367	4(5)	1(1.3)	Loss of airways
0	0	0	Paratracheal insertion
0	0	0	Pneumomediastinum
1	1(1.3)	0	Pneumothorax
1	1(1.3)	0	Subcutaneous emphysema
1	1(1.3)	0	Stoma infection
0.043*	16(20)	7(8.75)	Total rate of complications

SSPDT: Semi-surgical Percutaneous Dilatational Tracheostomy, CPDT: Conventional         Percutaneous Dilatational Tracheostomy

## Discussion

This study compared the semi-surgical percutaneous dilatational tracheostomy (SSPDT) with conventional percutaneous dilatational tracheostomy (CPDT). A total of 160 patients were randomly divided into two equal groups. One of the most important finding of this study was the duration of the procedure, which is shorter in MPDT group than CPDT group. The main reason for the short duration of the procedure in SSPDT group seems to be due to a wider incision of the skin (though not as much as the surgical method) and performing PDT by touching the trachea and seeing it directly. Also, the direct vision of the trachea and the short duration of the procedure caused the total complications in MPDT group to be less than CPDT group.

The most common reason for tracheostomy in the ICU is prolonged ventilatory support. Since the majority of the patients had CVAs in this study, the most common cause was a reduced level of consciousness. In a study entitled" A prospective randomized study comparing mini-surgical percutaneous dilatational tracheostomy with surgical and classical percutaneous tracheostomy" conducted in 2015 at Masih Daneshvari Hospital in Tehran by Hashemian et al.,the patients were initially divided into PDT and ST groups according to their indications, and the PDT group was then randomly divided into msPDT and cPDT groups. The duration of surgery was shorter and tracheostomy complications were fewer in the msPDT group compared to the cPDT group. The researchers concluded that msPDT is more suitable than cPDT (14). 

In our study, however, the patients with PDT contraindications were excluded. Despite the slight difference between the two studies in the randomization method used, the results were similar. The present study also found that SSPDT is a better method for tracheostomy than conventional PDT. Similarly, in Hashemian’s study, hypoxia occurred less frequently during the procedure in the SSPDT group compared to the conventional PDT group in the present study. The duration of the procedure was also shorter and the frequency of complications was lower in the SSPDT group compared to the CPDT group. Moreover, in our study, PDT was performed by the same person, which comprises another difference with Hashemian’s study.

In a study conducted on 207 patients entitled "Can intensive care physicians safely perform percutaneous dilatational tracheostomy", subcutaneous emphysema without pneumothorax occurred in one patient. Four of the patients underwent surgery again after their PDT. Early hemorrhage (in the first 48 hours after PDT) occurred in two patients, and delayed hemorrhage (ten days after PDT) occurred in one. Due to inadvertent decannulation, PDT turned into surgical tracheostomy in one patient. One incident of death was reported due to the paratracheal insertion of the tracheostomy tube. No signs of infection were observed at the tracheal site or in its surrounding tissue. (24) One case of infection at the tracheal site was observed in our study in the CPDT group. No pneumomediastinum or paratracheal insertion occurred. The frequency of complications in that study was similar to the frequency reported for the CPDT group in the present study, although there were fewer complications in the SSPDT group. 

In a study conducted in 2014 in Urmia, Iran, 60 patients were randomly divided into PDT and surgical tracheostomy group, and significant differences were observed between the two groups in terms of the duration of mechanical ventilation, the duration of the tracheostomy procedure (P=0.001) and the costs associated. No significant differences were observed between the two groups in terms of age, gender, duration of ICU stay and tracheostomy complications such as hemorrhage, stoma infection, subcutaneous emphysema or loss of airways. (8) This study compared surgical tracheostomy with PDT; however, this comparison was not possible in the present study due to the small number of patients undergoing surgical tracheostomy. However, the prevalence of complications in PDT was not different from our study.

Klancir et al. introduced a patient who had developed bilateral pneumothorax following PDT. They found no lesions in the patient’s trachea in the bronchoscopy and thus suggested that bronchoscopy was not a reliable tool for exposing tracheal damage developed during PDT. They believed that mild tracheal damage and high-airway-pressure mechanical ventilation had caused pneumothorax (16) In our study, one patient experienced subcutaneous emphysema and pneumothorax. Moreover, four patients (2.5%) experienced hypotension and four (2.5%) developed transient hypoxia.

In a retrospective study, Kuechler et al. investigated the complications of PDT in 289 patients with brain trauma and reported hypotension in three patients and transient hypoxia in two. They concluded that PDT is a safe procedure for these patients. Although their study was retrospective, it presented similar results in terms of the frequency of the complications (25). Decker et al. studied the safety of PDT in patients with multiple traumas and reported the frequency of complications as 37.4% and found bleeding to be the most frequent complication (26.3%). 

Fracture of tracheal cartilage occurred in 6% of the patients. Other complications reported included misplaced guidewire, hypotension and reduced arterial oxygen saturation. They concluded that PDT is a safe procedure for trauma patients (26). In our study, the frequency of complications was 20% in the CPDT group, which is similar to the study by Decker et al.; however, the overall frequency of complications was 19.4% in the two groups, which can be attributed to the less obvious complications in the SSPDT group (8.75%). 

In another retrospective study, Pattnaik et al. investigated the complications of PDT without bronchoscopy in 300 patients. They reported the overall frequency of complications as 8.6%, which is less than that reported in the present study (20%); however, considering the transverse cutaneous incision made (1.5-2cm), it was similar to that in the SSPDT group. The duration of the procedure was 3.5 minutes in the study, which is less than that reported in the our study (5.79 minutes). The main reason for this disparity may because the procedure was performed by a skilled person with more than three years of experience, but in our study, data were collected after a learning curve of about three months. Like the present study, the most common complication in that study was bleeding. Severe bleeding requiring transfusion occurred in two patients (0.66%) in the Kuechler et al.’s study. In our study, severe bleeding occurred in three patients (3.8%) but they did not need blood transfusion. In line with the present findings, tracheal stenosis had a frequency of 1.3% in that study (27).

In a cross-sectional study, Karimpour et al. investigated the complications of PDT in 184 patients using the Griggs method. The overall frequency of complications was 16.7%, the frequency of bleeding was 9.3%, puncture of the tracheal tube cuff1.6%, subcutaneous emphysema 1.1% and loss of airways 1.7%. The overall frequency of complications was the same as in the SSPDT group(16.7%) in the present study (28).

In conventional PDT, the cutaneous incision is small (less than 1 cm) and the needle is blindly inserted into the trachea; as a result, tracheal damage, insertion into the paratracheal space, tearing of the vessels surrounding the trachea and esophageal tearing are probable. Considering the 1.5-2-cm transverse incision made 1cm below the cricoid in the SSPDT group in the present study and considering that the subcutaneous tissue was pushed aside with the index figure to fully expose the tracheal ring, the frequency of complications, especially severe bleeding and loss of airways, is reported to be lower in this group than in the CPDT group.

The duration of the procedure was shorter in the SSPDT group than in the CPDT group. The complication rate was lower in the SSPDT group compared to the CPDT group. According to the findings of this study, SSPDT is safer than CPDT in ICU patients and is recommended to be used as an alternative to CPDT.

## Study Limitations:

Considering that PDT is widely performed in medical ICUs, and since patients with thyroid masses, cervical infections and emergency airway surgery are hospitalized in surgical ICUs, there were only two candidates for surgical tracheostomy, and to compare with SSPDT, surgical tracheostomy was therefore not possible. 

## Recommendations:

Future studies are recommended to conduct a comparative study of normal people and those with difficult airway anatomy (obese and short-necked) in terms of SSPDT complications.

## Financial Disclosure:

The authors declare no financial interests related to the material in the manuscript.
